# The Impact of Histologic Portal T-Cell Density on the Clinical Outcomes in Hepatic Graft-versus-Host Disease and Autoimmune Liver Diseases

**DOI:** 10.3390/diagnostics14161745

**Published:** 2024-08-12

**Authors:** Soon Kyu Lee, Sung-Soo Park, Silvia Park, Sung-Eun Lee, Byung-Sik Cho, Ki-Seong Eom, Yoo-Jin Kim, Hee-Je Kim, Chang-Ki Min, Seok-Goo Cho, Jong Wook Lee, Seok Lee, Younghoon Kim, Ji Won Han, Hyun Yang, Si Hyun Bae, Jeong Won Jang, Jong Young Choi, Seung Kew Yoon, Dong Yeup Lee, Sung Hak Lee, Jae-Ho Yoon, Pil Soo Sung

**Affiliations:** 1 Division of Gastroenterology and Hepatology, Department of Internal Medicine, Incheon St. Mary’s Hospital, College of Medicine, The Catholic University of Korea, Seoul 06591, Republic of Korea; blackiqq@catholic.ac.kr; 2 The Catholic University Liver Research Centre, College of Medicine, The Catholic University of Korea, Seoul 06591, Republic of Korea; tmznjf@catholic.ac.kr (J.W.H.); oneggu@naver.com (H.Y.); baesh@catholic.ac.kr (S.H.B.); garden@catholic.ac.kr (J.W.J.); jychoi@catholic.ac.kr (J.Y.C.); yoonsk@catholic.ac.kr (S.K.Y.); dyl15@naver.com (D.Y.L.); 3 Department of Hematology, Catholic Hematology Hospital and Leukemia Research Institute, Seoul St. Mary’s Hospital, College of Medicine, The Catholic University of Korea, Seoul 06591, Republic of Korea; sspark@catholic.ac.kr (S.-S.P.); silvia.park@catholic.ac.kr (S.P.); lee86@catholic.ac.kr (S.-E.L.); cbscho@catholic.ac.kr (B.-S.C.); dreom@catholic.ac.kr (K.-S.E.); yoojink@catholic.ac.kr (Y.-J.K.); cumckim@catholic.ac.kr (H.-J.K.); ckmin@catholic.ac.kr (C.-K.M.); chosg@catholic.ac.kr (S.-G.C.); jwlee@catholic.ac.kr (J.W.L.); leeseok@catholic.ac.kr (S.L.); 4 Department of Hospital Pathology, Seoul St. Mary’s Hospital, College of Medicine, The Catholic University of Korea, Seoul 06591, Republic of Korea; glabella85@gmail.com; 5 Division of Gastroenterology and Hepatology, Department of Internal Medicine, Seoul St. Mary’s Hospital, College of Medicine, The Catholic University of Korea, Seoul 06591, Republic of Korea; 6 Division of Gastroenterology and Hepatology, Department of Internal Medicine, Eunpyeong St. Mary’s Hospital, College of Medicine, The Catholic University of Korea, Seoul 06591, Republic of Korea

**Keywords:** GVHD, steroid, autoimmune hepatitis, primary biliary cholangitis, T cell

## Abstract

Hepatic graft-versus-host disease (GVHD) significantly impacts morbidity and mortality among allogeneic hematopoietic stem cell transplant recipients. However, the relationship between clinical and immunopathological phenotypes and their influence on clinical outcomes in hepatic GVHD is not well understood. In this study, we aimed to study the implications of portal T-cell infiltration on the clinical outcomes in hepatic GHVD and its similarities to autoimmune liver disease. We analyzed 78 patients with biopsy-confirmed hepatic GVHD (*n* = 38) or autoimmune liver disease (*n* = 40) between 2016 and 2021. The cholestatic variant was defined by an R-value < 2.0, based on the ratio of alanine aminotransferase to alkaline phosphatase. The primary outcome was the biochemical response at 4 (early) and 8–12 (late) weeks after corticosteroid treatment. In hepatic GVHD patients, the hepatitic variant (*n* = 19) showed greater CD3^+^ T-cell infiltration than the cholestatic variant (*n* = 19; *p* < 0.001). No significant differences were observed in the infiltration of CD20^+^, CD38^+^, or CD68^+^ cells. The hepatitic variant had significantly better early and late responses and higher liver-related event-free survival than the cholestatic variants (*p* < 0.05). Concerning autoimmune liver diseases, the autoimmune hepatitis (AIH) group had significantly more portal T-cell infiltration and better treatment responses than the primary biliary cholangitis (PBC) group. In conclusion, higher portal T-cell infiltration may be associated with better clinical outcomes in patients with hepatic GVHD. Additionally, this study highlights similarities in portal T-cell infiltration and treatment response patterns between AIH and the hepatitic variant, as well as PBC and the cholestatic variant.

## 1. Introduction

Allogenic hematopoietic stem cell transplantation (Allo-HSCT), an intensive treatment for hematological malignancies and genetic diseases, is conducted in over 25,000 cases annually [1]. Approximately 60% of allo-HSCT recipients develop chronic graft-versus-host disease (GVHD), which can affect several organs, including the skin, eyes, gastrointestinal tract, lungs, and liver [1]. Notably, chronic GVHD is one of the leading cause of long-term morbidity and mortality following allo-HSCT [1,2].

The liver is one of the organs most frequently affected after allo-HSCT, and the development of hepatic GVHD is associated with a poor prognosis in allo-HSCT recipients [3]. Clinically, hepatic GVHD can manifest in different phenotypes: first, with notably elevated alkaline phosphatase (ALP), and bilirubin levels, termed the cholestatic variant; second, with marked elevations in aspartate transaminase (AST) and alanine transaminase (ALT), termed the hepatitic variant [4,5]. Histopathologically, hepatic GVHD is characterized by portal inflammation, lobular hepatitis, and damage to the bile duct epithelium [6]. However, the association between clinical and immuno-pathological phenotypes and their impact on the clinical outcomes of hepatic GVHD remains unclear.

Because hepatic GVHD affects the mortality and morbidity of allo-HSCT recipients, several studies have sought to identify prognostic factors for hepatic GVHD. The presence of lung or gastrointestinal involvement is a well-known risk factor for poor outcomes in patients with hepatic GVHD [7]. Moreover, severe lobular activity accompanied by mild hepatocyte ballooning in the liver has been suggested as a significant factor for non-relapse mortality [8]. A recent study demonstrated that an infiltration of T helper 17 (Th17) cells, in conjunction with an increased Th17/regulatory T (Treg) cells ratio, was observed in the livers of patients with chronic hepatic GVHD [9]. Furthermore, donor T follicular helper cells are believed to play a role in the development of chronic hepatic GVHD [10]. Given the notable pathophysiological influence of infiltrated T-cells in the livers of patients with chronic hepatic GVHD, their impact on the clinical outcomes should be clarified to enhance these patient’s prognosis.

Similar to the pivotal role of T cells in hepatic GVHD, autoimmune liver diseases such as autoimmune hepatitis (AIH) and primary biliary cholangitis (PBC) arise from an imbalance in self-antigen tolerance, leading to the activation of autoreactive T cells [11]. Specifically, AIH is a chronic liver disease that targets and damages hepatocytes, while PBC affects small- to medium-sized intrahepatic bile ducts [11,12]. These observations suggest a potential similarity in histological characteristics between autoimmune liver diseases and GVHD, an area that has not been extensively explored.

To address these gaps in knowledge, we examined the impact of the histological features of hepatic GVHD, with a particular focus on portal T-cell density, on clinical outcomes following allo-HSCT. Additionally, we analyzed the histopathology of patients diagnosed with AIH or PBC, and evaluated its influence on treatment outcomes. Finally, we compared the histopathological characteristics and their implications for clinical outcomes between hepatic GVHD and autoimmune liver diseases.

## 2. Results

### 2.1. Baseline Characteristics of Entire Population

Among the 38 patients with hepatic GVHD included in the study, 19 were classified as having the hepatitic variant, and the remaining 19 were identified as having the cholestatic variant (Table 1). The median age of the patients was 47 years, and males constituted 52.6% (*n* = 20) of the cohort. The majority (81.6%) of the patients, irrespective of the variant, underwent allo-HSCT due to acute myeloid leukemia (AML), with no notable differences observed between the groups. No significant differences were found in the transplant source, donor type, ABO mismatch, HLA match, or conditioning regimen used for HSCT. However, in terms of GVHD prophylaxis, the cholestatic variant group showed a higher frequency of treatment with cyclosporine/methotrexate than the hepatitic variant group (*p* = 0.03). The median time from post-transplant to diagnosis of hepatic GVHD via liver biopsy was 6.0 (range, 3.0–61.1) months.

### 2.2. Characteristics of Hepatitic and Cholestatic Variants

In terms of simultaneously involved organs, GI tract was the most commonly involved organ without significant differences between hepatitic and cholestatic variant (Table 2). Upon examination of laboratory results, patients with the hepatitic variant displayed lower total bilirubin levels and higher ALT levels than those with the cholestatic variant, as shown in Supplementary Table S1. The levels of albumin, AST, ALP, GGT, creatinine, INR, and MELD scores were not significantly different between the two groups. Histopathologically, the necro-inflammatory scores and fibrosis stages were similar between the groups. Regarding treatment for hepatic GVHD, both groups received an average prednisolone dose of approximately 50 mg/day.

### 2.3. Comparison of Portal T-Cell Infiltration between the Two Groups

**Figure 1:**
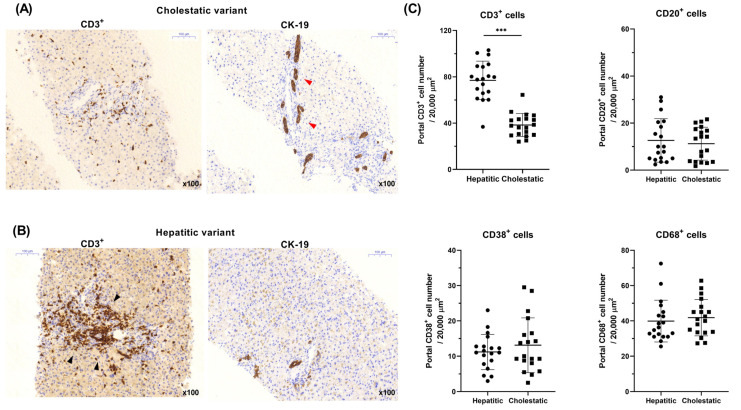
Representative liver histology of (**A**) cholestatic and (**B**) hepatitic variants (red arrow, bile duct damage and proliferation; black arrow, portal T-cell infiltration) and (**C**) comparison of immunohistochemistry according to variants, all presented in a x100 microscopic field. *** *p* < 0.001.

As depicted in Figure 1A,B, patients with the cholestatic variant exhibited a lower number of infiltrated CD3^+^ cells (T cells) than those with the hepatitic variant. However, the cholestatic variant showed a more pronounced CK-19 expression. The concentration of infiltrated CD3^+^ cells in the portal area for the hepatitic variant group was significantly elevated (76.96 vs. 38.38 cells/20,000 μm^2^; *p* < 0.001) compared to that in the cholestatic variant group. However, no significant differences were observed in other cell types, such as CD20^+^, CD38^+^, and CD68^+^, between the two groups (Figure 1C), indicating that the density of portal T-cell infiltration is a distinctive feature that differentiates the two groups.

### 2.4. Comparison of Clinical Outcomes between Two Groups-Response, Relapse, Liver-Related Event-Free Survival (EFS)

**Figure 2:**
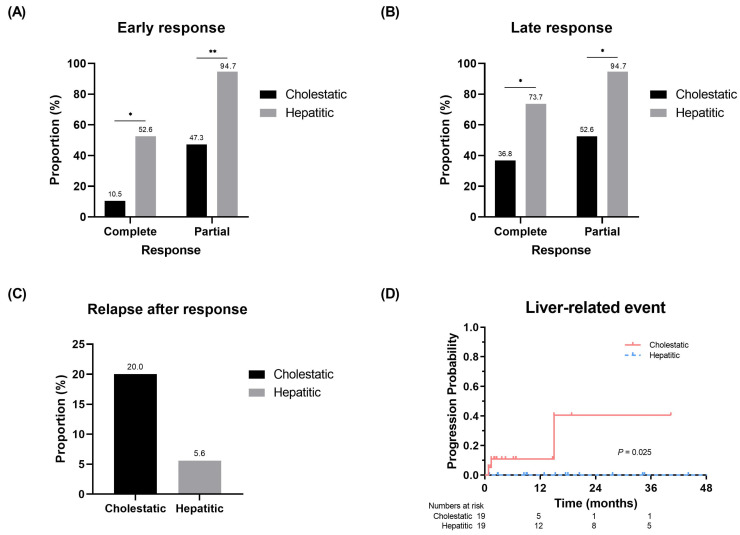
Comparison of (**A**,**B**) treatment response for steroid therapy and (**C**) relapse in patients with late response between cholestatic and hepatitic variants. (**D**) Liver-related event-free survival curves according to cholestatic and hepatitic variants. * *p* < 0.05, ** *p* < 0.01.

To evaluate the potential association between portal T-cell infiltration and clinical outcomes in hepatic GVHD, we analyzed the treatment response, relapse, and liver-related EFS according to the variants. For the early response, the hepatitic variant group demonstrated a significantly higher proportion of complete (52.6% vs. 10.5%, respectively; *p* < 0.05) and partial (94.7% vs. 47.3%, respectively; *p* < 0.01) responses compared to the cholestatic variant group (Figure 2A). These results were similar for the late response, with a higher response rate observed in the hepatitic variant compared to the cholestatic variant (complete response rate, 73.7% vs. 36.8%, respectively, *p* < 0.05; partial response rate, 94.7% vs. 52.6%, *p* < 0.05; Figure 2B). Moreover, the proportion of relapse after response was marginally higher in the cholestatic variant (Figure 2C).

Regarding liver-related events, three patients initially classified into the cholestatic variant group experienced disease progression, resulting in liver failure (*n* = 1) or death (*n* = 2), with a median follow-up of 8.8 (0.8–62.5) months. There were no liver-related events in the hepatitic variant, leading to a significantly higher liver-related EFS rate (*p* = 0.025; Figure 2D).

To further validate the association between portal T-cell infiltration and the outcomes of chronic hepatic GVHD, we evaluated clinical outcomes in relation to the degree of portal T-cell infiltration. As depicted in Supplementary Figure S1, patients with high portal T-cell infiltration (the high group) demonstrated significantly better complete and partial responses compared to those with low portal T-cell infiltration (the low group) (*p* < 0.05 for both). The proportion of relapse after response was marginally higher in the low group. Additionally, mirroring the results observed between the hepatitic and cholestatic groups, the high group showed a significantly higher liver-related event-free survival (EFS) rate (*p* = 0.036), with no liver-related events observed in the high group (Supplementary Figure S1D).

### 2.5. Pathological Changes in a Patient with Cholestatic Variant Who Underwent Liver Transplantation (LT)

**Figure 3:**
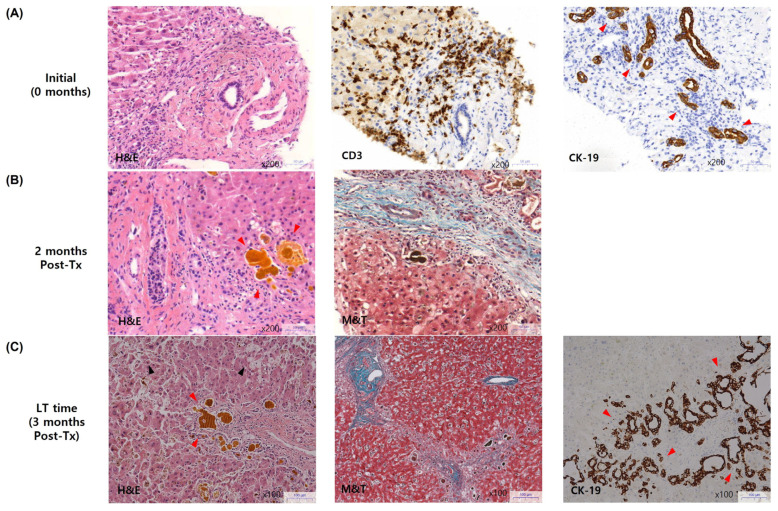
The serial changes of immunohistopathologic findings in the liver of a patient who underwent transplantation due to hepatic failure caused by chronic hepatic GVHD with cholestatic variants, presented in either ×100 or ×200 microscopic field. Serial liver tissue samples were obtained (**A**) at the time of diagnosed with chronic hepatic GVHD (red arrow, bile duct damage), (**B**) two months after initiating GVHD treatment (red arrow, cholestasis), and (**C**) during liver transplantation (red arrow, cholestasis and bile duct proliferation; black arrow, hepatocellular necrosis). GVHD, graft-versus-host disease; LT, liver transplantation; Tx, treatment.

Among the three patients who experienced liver-related events, one diagnosed with the cholestatic variant of hepatic GHVD underwent LT due to liver failure. The patient was a 54-year-old male who initially presented with chronic hepatic GVHD 6 months post-HSCT for AML. At diagnosis, the patient’s laboratory values were an ALT of 126 U/L, an ALP of 186 U/L (resulting in an R-value of 0.68), and a total bilirubin level of 5.75 mg/dL. Histopathologically (Figure 3A), moderate lobular damage and periportal activity were observed, along with a mild density of CD3^+^ cells and bile duct damage.

Despite treatment with steroids, MMF, high-dose UDCA, and the later addition of tacrolimus, the patient’s liver function further deteriorated (total bilirubin, 16.73 mg/L; ALT, 32 U/L, and ALP, 96 U/L). Liver tissue from a transjugular intrahepatic liver biopsy performed 2 months after treatment revealed advanced cholestasis, bile duct loss, and portal fibrosis (Figure 3B). Three months after the initial diagnosis, liver sonography indicated ascites and portal hypertension with a Child–Pugh score of 10 (class C) and a MELD score of 24. Consequently, he underwent living donor LT from his brother, who had also been a donor for HSCT. The explanted liver showed severe hepatocellular necrosis and cholestasis with bile duct proliferation (Figure 3C), indicative of hepatic GVHD progression.

### 2.6. A Comparison of Clinical and Pathological Findings in Autoimmune Liver Disease

Among the 40 patients with autoimmune liver disease, those with AIH were older on average than those with PBC (65.0 vs. 53.0 years, respectively; *p* = 0.013). The AIH group also had higher total bilirubin, AST, and ALT levels than the PBC group (*p* < 0.05 for all), whereas ALP and GGT levels were significantly higher in the PBC group (*p* < 0.05) (Supplementary Table S2).

**Figure 4:**
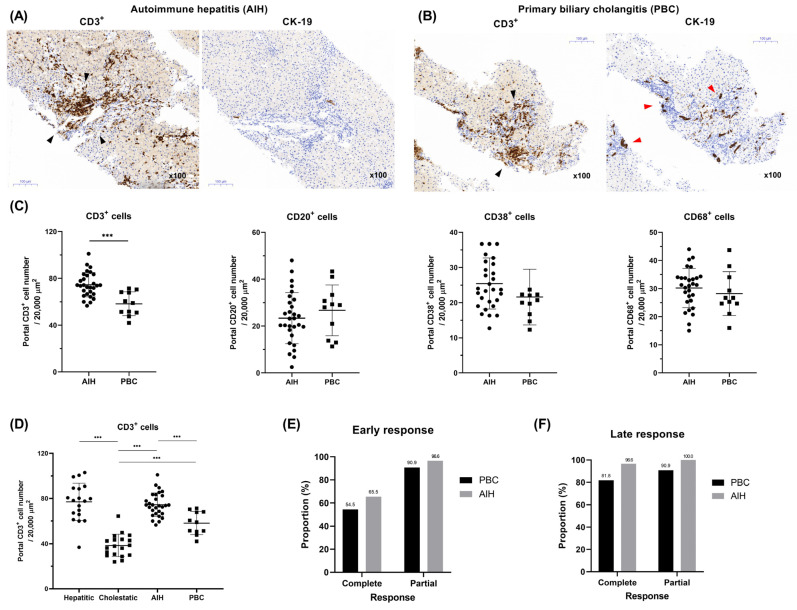
Representative liver histology of (**A**) AIH and (**B**) PBC (black arrow, portal T-cell infiltration; red arrow, bile duct damage and proliferation) and (**C**,**D**) comparison of immunohistochemistry between AIH, PBC, and GVHD. (**E**,**F**) Comparison of treatment response between AIH and PBC. AIH, autoimmune hepatitis; PBC, primary biliary cholangitis; GVHD, graft-versus-host disease. *** *p* < 0.001.

Histopathologically, the necroinflammatory score (NIS) score was significantly higher in the AIH group than in the PBC group, with no notable differences in fibrosis levels. Immunohistochemical analysis revealed significant differences between AIH and PBC in terms of CD3^+^ cell infiltration and bile duct damage (Figure 4A,B). Specifically, the AIH group exhibited more extensive infiltration of CD3^+^ cells (T cells) in the portal area than the PBC group (median, 73.8 vs. 59.0, respectively, *p* = 0.001; Figure 4C). However, the infiltration levels of other immune cells, including CD20^+^, CD38^+^, and CD68^+^ cells, did not differ significantly between groups (Figure 4C).

In conjunction with hepatic GVHD, the density of infiltrated CD3^+^ cells in the periportal area were significantly higher in the hepatitic and AIH groups, followed by that in the PBC and cholestatic groups (Figure 4D). Meanwhile, CD20^+^ and CD38^+^ cells were significantly higher in the periportal area of the AIH and PBC groups compared to both the hepatitic and cholestatic groups, while a reversed pattern was observed for CD68^+^ cells, which were higher in the hepatitic and cholestatic groups than in the AIH and PBC groups (Supplementary Figure S2). In terms of treatment response, the AIH group exhibited a marginally higher response than the PBC group (Figure 4E,F), whereas the response rates of both groups surpassed those of the hepatitic and cholestatic variant groups.

## 3. Discussion

This study examined the histopathological variations in hepatic GVHD, with particular emphasis on the types of immune cells infiltrating the portal area. Our detailed analysis highlighted significant disparities in the density of portal T-cell infiltration between hepatitic and cholestatic variants. Notably, the hepatitic variant group, characterized by higher portal T-cell infiltration, showed more favorable clinical outcomes, including better treatment responses and liver-related EFS. In contrast, the cholestatic variant group displayed lower treatment responsiveness and, in some cases, progressed to hepatic failure, necessitating LT. Additionally, when compared to autoimmune liver diseases, the hepatitic and AIH groups demonstrated significantly higher densities of infiltrated T-cells in the periportal area, followed by the PBC and cholestatic groups. This trend was similar to that observed for treatment responses. Our study is the first to comprehensively elucidate the variations in portal T-cell infiltration among patients with hepatic GVHD, its association with clinical outcomes, and to draw relevant comparisons with autoimmune diseases.

Interestingly, our immunohistochemical analysis revealed a notable difference in portal T-cell infiltration density between hepatitic and cholestatic variants of hepatic GVHD. Although the pathophysiology of this condition remains incompletely understood, emerging evidence suggests a pivotal role for T-cells in the development of chronic GVHD. Studies in mouse models indicate that the expansion of Th1 and Th17 cells, coupled with the depletion of Treg cells, contributes to chronic GVHD onset [13,14]. Additionally, increased levels of Th17 cells, their related cytokines, and the Th17/Treg ratio have been documented in patients with chronic GVHD [9,15,16], corroborating our findings. Recently, a study analyzing cytokines in patients with abnormal liver function after allo-HSCT demonstrated distinct differences in cytokine elevations according to the pattern of liver injury [17]. A hepatocellular pattern of liver injury shows elevated serum amyloid A, interleukin (IL)-15, and Transforming growth factor (TGF)-α, while a cholestatic pattern exhibits higher levels of IL-6, IL-10, IL-13, and interferon (IFN)-γ [17]. These findings suggest that distinct differences in the pathogenesis of liver injury may exist according to its pattern, substantiating our observations. Indeed, the variations in portal T-cell density observed in our study suggest potentially distinct underlying pathogenic mechanisms that drive the development of each hepatic GVHD variant.

Notably, our study found a potential association between the portal T-cell infiltration density and clinical outcomes. The hepatitic variant, characterized by higher portal T-cell infiltration, demonstrated significantly better early and late treatment responses than the cholestatic variant, which exhibited lower T-cell infiltration. Although we could not determine the specific types of infiltrated portal T-cells, it is plausible that most of these cells are inflammatory, considering previous findings of high Th17 cell counts in patients with chronic hepatic GVHD [9]. From the perspective of steroid treatment, we have previously reported that steroids can facilitate the recovery of patients with drug-induced liver injury (DILI) by resolving activated liver-infiltrating CD3^+^ and CD8^+^ T cells [18]. Given this effect of steroids, the enhanced response in the hepatitic variant could be attributed to the abundant portal T-cell infiltration, primarily comprising inflammatory cells, thereby rendering steroid therapy more effective. In contrast, the cholestatic variant, characterized by lower portal T-cell infiltration and fewer inflammatory cells, may exhibit a reduced response to steroid therapy. Therefore, the increased portal T-cell density in the hepatitic variant could lead to better steroid responses, reducing the severity of chronic hepatic GVHD, potentially resulting in reduced relapses and better liver-related EFS. Consequently, our findings suggest that high portal T-cells infiltration may be a favorable prognostic indicator in patients with chronic hepatic GVHD. Conversely, patients with low T-cell infiltration, due to their poor treatment responses, may need to consider alternative or second-line treatments early on, including those with different therapeutic mechanisms such as inhibiting JAK pathway [19].

Indeed, three patients initially classified into the cholestatic variant group, which is marked by low portal T-cell infiltration, ultimately experienced liver-related events. Through swift decision-making and the availability of a suitable donor, one of these patients was successfully treated with a living donor LT from his brother, who had also been his HSCT donor. Impressively, this patient survived without any complications for three years following LT. Histopathological examination of the explanted liver indicated that chronic hepatic GVHD can precipitate severe hepatocyte necrosis and notable bile duct proliferation, particularly in severe cases that are unresponsive to treatment. This observation indicates that timely LT may be considered a means to improve outcomes in patients with cholestatic variants of chronic hepatic GVHD who do not respond to conventional therapies.

Regarding autoimmune liver diseases, including AIH and PBC, we noted differences in portal T-cell infiltration and treatment responsiveness between the two groups. Although PBC can display moderate infiltration of portal T-cells, AIH exhibits significantly higher infiltration, paralleling better treatment responses. These findings suggest a notable resemblance between AIH and the hepatitic variant, as well as between PBC and the cholestatic variant. Given that the hallmark of AIH is the activation of autoreactive T cells due to lose tolerance [11,20], steroids are the main treatment for AIH. They work by enhancing Treg cell functions and restoring immune homeostasis [21,22]. This mirrors the favorable response observed in the hepatitic variant of chronic hepatic GVHD, potentially explained by the pronounced T-cell infiltration seen in both diseases via Treg induction and immune stabilization. Indeed, a recent study demonstrated a predominant infiltration of CD8^+^ T cells along with an absence of Treg cell activation in patients with hepatic GVHD [23]. Moreover, we have previously documented Treg expansion in steroid responders with severe alcohol-associated liver diseases [24]. These results lend support to our hypothesis that steroid therapy ameliorates the hepatitic variant of hepatic GVHD by enhancing Treg cells. Meanwhile, hepatocyte damage in PBC, primarily a consequence of cholestasis and concentrated bile acids, does not respond effectively to steroid therapy [25,26,27]. This might also explain the diminished treatment response observed in the cholestatic variant of chronic hepatic GVHD observed in our study. These insights underline the necessity for further investigation into the underlying pathophysiology and treatment modalities of chronic hepatic GVHD to enhance patient outcomes and tailor therapeutic approaches.

Our study had several limitations. First, the study was retrospective in nature. Second, the sample size was relatively small. Third, investigating the pathophysiological mechanisms underlying hepatic GVHD remains challenging. Fourth, we could not evaluate the specific type of infiltrated T-cells in portal area. However, it is important to recognize that collecting and analyzing liver histopathological data for hepatic GVHD is arduous, because of the rarity of the disease and the inherent difficulties in performing liver biopsies in these patients. Despite these challenges, our study successfully included a considerable number of patients, facilitating comprehensive histopathological analysis of hepatic GVHD. To the best of our knowledge, this is the first study to highlight the significance of portal T-cell infiltration in treatment response and clinical outcomes by examining liver tissue IHC. To further validate and overcome these limitations, future research should involve evaluating the specific types of T-cells infiltrating in hepatic GVHD and examining these results in an in vivo model, including a GVHD mouse model. Meanwhile, we also evaluated the histopathology of autoimmune liver disease, drawing parallels and postulating potential mechanisms for the differing treatment responses between the hepatitic and cholestatic variants of hepatic GVHD. Although the pathogenesis of PBC remain unclear with diverse severity, we intriguingly identified resemblances between the hepatitic variant and AIH, as well as between the cholestatic variant and PBC. These observations provide insights into the underlying mechanism and help explain the differing treatment responses among these variants. Considering the limited number of patients with PBC included in our study, further research with larger patient cohort is warranted to validate our findings.

## 4. Conclusions

In conclusion, our study revealed that the hepatitic variant, characterized by higher portal T-cell infiltration, demonstrated significantly better clinical outcomes compared to the cholestatic variant, which exhibited lower portal T-cell infiltration. This finding provides insights into the differing profiles of portal T-cell infiltration in hepatitic and cholestatic variants of hepatic GVHD, emphasizing the impact of these variations on clinical outcomes. Consequently, assessing portal T-cell infiltration in patients with hepatic GVHD may assist in determining the most appropriate treatment drugs and predicting patient outcomes. In patients with high portal T-cells infiltration, steroid therapy could be sufficient to improve clinical outcomes, while those with low T-cell infiltration may need to consider alternative or second-line treatments early on. If performing a liver biopsy is not feasible, categorizing patients into hepatitic and cholestatic groups might help to assume the portal T-cell infiltration and thus potentially predict their response to steroid therapy. Furthermore, our findings highlight the similarities in portal T-cell infiltration and treatment responses between the hepatitic variant and AIH, as well as between the cholestatic variant and PBC, warranting further validation studies.

## 5. Materials and Methods

### 5.1. Study Population

**Figure 5:**
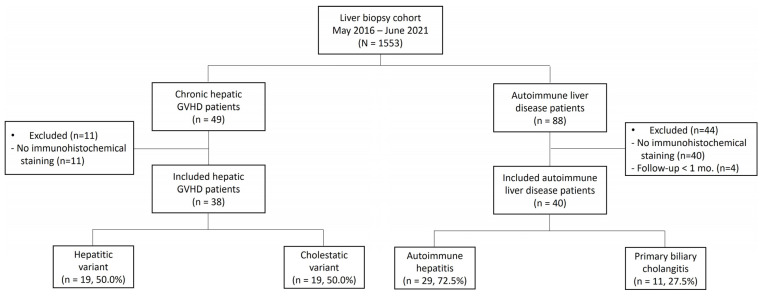
Study flow chart.

A total of 1553 patients who underwent liver biopsies between May 2016 and June 2021 at Seoul St. Mary’s Hospital, The Catholic University of Korea were consecutively screened for eligibility. Of these, 49 patients with hepatic GVHD and 88 patients with autoimmune liver disease were enrolled in the study. Among the 49 patients with hepatic GVHD, 11 were excluded because of the absence of immunohistochemical staining in the liver tissue; the remaining 38 patients were included in the final analysis. Meanwhile, of the 88 patients with autoimmune liver diseases, 44 were excluded for the following reasons: patients without immunohistochemical staining of the liver (*n* = 40) and patients with a follow-up of less than one month (*n* = 4). Finally, 40 patients with autoimmune liver disease patients were included in the final analysis (Figure 5). This study adhered to the principles of the Declaration of the Helsinki and was approved by the Institutional Review Board of the Seoul St. Mary’s Hospital, The Catholic University of Korea (KC20WISI0394).

### 5.2. Diagnosis and Classification

Hepatic GVHD was diagnosed based on recent guidelines [28,29], which incorporate both laboratory findings–such as elevated serum levels of ALT, ALP, and bilirubin–and histopathologic findings, including portal inflammation, lobular injury, and bile duct damage [6]. Before confirming the diagnosis of hepatic GVHD, other differential diagnoses such as viral hepatitis and drug-induced liver injury were ruled out. For histopathological evaluation of hepatic GVHD, two expert pathologists (SH Lee and Y Kim) reviewed the liver specimens. Following the diagnosis, hepatic GVHD cases were categorized into two groups based on their liver enzyme elevation patterns: cholestatic variant, patients with an R-value < 2.0, determined by the ratio of ALT to ALP; hepatitic variant, patients who had an R-value ≥ 2 at the time of their chronic hepatic GVHD diagnosis.

Diagnoses of AIH and PBC were made following the updated guidelines [30,31,32,33]. For AIH, both simplified and revised diagnostic criteria were utilized. PBC was diagnosed by combining various indicators, including elevated ALP, presence of anti-mitochondrial antibody, and histologic findings consistent with PBC. The diagnoses of both autoimmune liver diseases were confirmed after excluding other diseases, including alcohol-related disease, viral hepatitis, and drug-induced liver injury. Histological evaluation of the liver for AIH and PBC was performed by two expert pathologists (SH Lee and Y Kim).

### 5.3. Clinical, Laboratory, and Histopathological Assessment

Upon the diagnosis of hepatic GVHD, various clinical characteristics of the patients were assessed. This included age, sex, underlying hematological disease necessitating HSCT, transplant source, donor type, ABO and HLA compatibility, conditioning regimen, and GVHD prophylaxis. Additionally, laboratory findings for patients diagnosed with either hepatic GVHD or autoimmune liver disease were collated, including levels of total bilirubin, albumin, AST, ALT, ALP, gamma-glutamyl transferase (GGT), creatinine, international normalized ratio (INR), and Model for End-stage Liver Disease (MELD) scores at the time of diagnosis.

To evaluate liver histopathology in hepatic GVHD and autoimmune liver diseases, biopsy specimens were deemed suitable if they were >10 mm in length and/or contained at least 12 portal areas. Once these were routinely fixed and stained, the presence and extent of portal and lobular inflammation and bile duct damage were assessed. To determine the types of immune cells infiltrating the portal area, immunohistochemical (IHC) staining was conducted using CD3, CD20, CD38, and CD68 markers to detect T-cells, B-cells, plasma cells, and macrophages, respectively. The number of positively stained cells was counted and averaged across five portal areas per 20,000 μm^2^. CK-19 staining was performed to analyze bile duct damage in the liver.

### 5.4. Treatment and Follow-Up

For patients diagnosed with hepatic GVHD, treatment was administered in line with established guidelines, comprising prednisolone (1 mg/kg/day) with or without calcineurin inhibitors (CNI), mycophenolate mofetil, and ursodiol (UDCA) [6,29]. A complete biochemical response was gauged using the Paris II criteria, which entailed ALP and AST levels below 1.5 times the upper limit of normal (ULN) and a total bilirubin level less than 1 mg/dL [27,34]. Partial response was defined as a reduction in the variables outlined by the Paris II criteria without fully meeting them. Relapse after a response was defined as an increase in liver enzyme levels after achieving at least a partial response. After initiating treatment, the patients underwent follow-up every 2–4 weeks, and laboratory tests, including liver function assessments, were conducted at every visit.

Based on treatment guidelines [30,31,32,33], patients with AIH were typically administered prednisolone (20–40 mg/day) with or without azathioprine (50–150 mg/day) as first-line therapy, whereas those with PBC received a high dose of UDCA (13–15 mg/kg/day). For AIH, a complete biochemical response was characterized by the normalization of AST, ALT, and immunoglobulin G (IgG) levels. For PBC, a complete response was determined by the achievement of the Paris II criteria [30,31,32]. Similar to hepatic GVHD, a partial response was defined as a partial reduction in the parameters outlined in the response criteria for each disease. After starting treatment, the patients underwent regular follow-up every 2–4 weeks, and laboratory tests were conducted during each visit.

### 5.5. Endpoints

The primary endpoint involved comparing the concentrations of infiltrated immune cells (CD3^+^, CD20^+^, CD38^+^, and CD68^+^ cells) and CK-19 positivity in both the hepatitic and cholestatic variants of hepatic GVHD as well as between AIH and PBC. Additionally, we assessed biochemical responses at 4 (early) and 8–12 (late) weeks post-treatment in these groups. Biochemical responses were categorized as complete, partial, or no response, as previously defined in the treatment section.

Secondary outcomes included comparisons of post-response relapse rates, baseline characteristics, and the liver-related EFS according to the variants in hepatic GVHD. Liver-related events were defined as liver failure or LT. Moreover, we investigated the clinical course and pathological changes in patients with liver-related events.

### 5.6. Statistical Analysis

Baseline characteristics of the patients were presented as mean ± standard deviation or median (range) for quantitative variables and as counts (%) for categorical variables, whichever was appropriate. Comparisons between the two groups were performed using a Student’s *t*-test or Mann–Whitney U test for continuous variables and the chi-square test or Fisher’s exact test for categorical variables, as appropriate. Kaplan–Meier analysis was used to estimate liver-related EFS. A *p*-value < 0.05 was considered statistically significant. All statistical analyses were conducted using R software (version 4.3.1; http://cran.r-project.org).

## Figures and Tables

**Table 1:** Baseline characteristics of entire population.

Variables	Total (*N* = 38)	Hepatitic Variant (*n* = 19)	Cholestatic Variant (*n* = 19)	*p*-Value
Age, years	47 (19–65)	43 (19–55)	49 (23–65)	0.052
Male sex (*n*,%)	20 (52.6%)	9 (47.4%)	11 (57.9%)	0.745
Diagnosis (*n*,%)				0.237
AML	31 (81.6%)	16 (84.2%)	15 (78.9%)	
ALL	2 (5.3%)	2 (10.5%)	0 (0.0%)	
MDS	1 (2.6%)	0 (0.0%)	1 (5.3%)	
Lymphoma	1 (2.6%)	0 (0.0%)	1 (5.3%)	
CML	2 (5.3%)	0 (0.0%)	2 (10.5%)	
MPD	1 (2.6%)	1 (5.3%)	0 (0.0%)	
Transplant source (*n*,%)				0.486
PBSCs	36 (94.7%)	17 (89.5%)	19 (100%)	
CB	2 (5.3%)	2 (10.5%)	0 (0.0%)	
Donor type (*n*,%)				0.100
Related	22 (57.9%)	8 (42.1%)	14 (73.7%)	
Unrelated	16 (42.1%)	11 (57.9%)	5 (26.3%)	
Sex mismatch (*n*,%)				1.000
Match	17 (44.7%)	8 (42.1%)	9 (47.4%)	
Mismatch	21 (55.3%)	11 (57.9%)	10 (52.6%)	
ABO mismatch (*n*,%)				0.882
Match	26 (68.4%)	12 (63.2%)	14 (73.4%)	
Minor mismatch	6 (15.8%)	4 (21.1%)	2 (10.5%)	
Major mismatch	6 (15.8%)	3 (15.8%)	3 (15.8%)	
Median Post-transplant time of biopsy (months, range)	6.0 (3.0–61.1)	4.8 (3.0–61.1)	6.1 (3.0–36.4)	0.759

AML, acute myeloid leukemia; ALL, acute lymphoblastic leukemia; MDS, myelodysplastic syndrome; CML, chronic myeloid leukemia; MPD, myeloproliferative disorder; PBSCs, peripheral blood stem cell; CB, cord blood.

**Table 2:** Involved organs among patients with hepatic GVHD.

Variables	Total (*N* = 38)	Hepatitic Variant (*n* = 19)	Cholestatic Variant (*n* = 19)	*p*-Value
Other organ involved (*n*,%)				0.324
None	5 (13.2%)	1 (5.3%)	4 (21.1%)	
Skin	7 (18.4%)	4 (21.1%)	3 (15.8%)	
GI tract	13 (34.2%)	8 (42.1%)	5 (26.3%)	
Lung	5 (13.2%)	4 (21.1%)	1 (5.3%)	
Skin and GI tract	4 (10.5%)	1 (5.3%)	3 (15.8%)	
Eyes	4 (10.5%)	1 (5.3%)	3 (15.8%)	

GI, gastrointestinal.

## Data Availability

Data are unavailable due to ethical restriction.

## Supplementary Materials

The following supporting information can be downloaded at: https://www.mdpi.com/article/10.3390/diagnostics14161745/s1, Table S1: Laboratory, histopathologic findings, and treatments at the time of liver biopsy between hepatitic and cholestatic variants; Table S2: Baseline characteristics and Laboratory, histopathologic findings and treatments between autoimmune hepatitis and primary biliary cholangitis; Figure S1: Comparison of (A, B) treatment responses to steroid therapy and (C) relapse rates in patients with late responses, between the high portal T-cell infiltration group (the high group) and the low portal T-cell infiltration group (the low group). (D) Liver-related event-free survival (EFS) curves according to the high and low groups. * *p* < 0.05, ** *p* < 0.01. Figure S2: Comparison of immunohistochemistry, including (A) CD20, (B) CD38, and (C) CD68, between AIH, PBC, and GVHD. AIH, autoimmune hepatitis; PBC, primary biliary cholangitis; GVHD, graft-versus-host disease. * *p* < 0.05, ** *p* < 0.01, *** *p* < 0.001.
