# Supplementary material for: The Impact of Histologic Portal T-Cell Density on the Clinical Outcomes in Hepatic Graft-versus-Host Disease and Autoimmune Liver Diseases
Source: Diagnostics (Basel). 2024 Aug 12;14(16):1745. doi: 10.3390/diagnostics14161745 (PMC11353783; doi:10.3390/diagnostics14161745)

Supplementary Materials

The Impact of Histologic Portal T-Cell Density on the Clinical Outcomes in Hepatic Graft-versus-Host Disease and Autoimmune Liver Diseases

Soon Kyu Lee [1,2], Sung-Soo Park [3], Silvia Park [3], Sung-Eun Lee [3], Byung-Sik Cho [3], Ki-Seong Eom [3], Yoo-Jin Kim [3], Hee-Je Kim [3], Chang-Ki Min [3], Seok-Goo Cho [3], Jong Wook Lee [3], Seok Lee [3], Younghoon Kim [4], Ji Won Han [2,5], Hyun Yang [2,6], Si Hyun Bae [2,6], Jeong Won Jang [2,5], Jong Young Choi [2,5], Seung Kew Yoon [2,5], Dong Yeup Lee [2,5], Sung Hak Lee [4,*], Jae-Ho Yoon [3,*] and Pil Soo Sung [2,5,*]

Table of contents

Supplementary Table S1……………………………………………………………………………2

Supplementary Table S2……………………………………………………………………………3

Supplementary Figure S1…………………………………………………………………………4

Supplementary Figure S2…………………………………………………………………………5

Supplementary Table S1. Laboratory, histopathologic findings, and treatments at the time of liver biopsy between hepatitic and cholestatic variants

Variables	Total (N=38)	Hepatitic variant (n=19)	Cholestatic variant (n=19)	*P*-value
Laboratory findings				
Total bilirubin (mg/dL)	3.2 ± 3.7	2.2 ± 3.6	4.3 ± 3.5	0.014
Albumin (g/dL)	3.4 ± 0.4	3.5 ± 0.4	3.4 ± 0.4	0.314
AST (U/L)	217 (70-1145)	207 (108-1145)	250 (70-849)	0.704
ALT (U/L)	394 (53-1125)	436 (135-1125)	240 (53-1065)	0.077
ALP (U/L)	208 (45-672)	210 (45-376)	206 (94-672)	0.781
GGT (U/L)	845 (89-2000)	900 (106-2000)	664 (89-2000)	0.793
Creatinine (mg/dL)	0.86 ± 0.47	0.87 ± 0.4	0.85 ± 0.55	0.599
INR	1.02 ± 0.14	1.01 ± 0.11	1.04 ± 0.16	0.737
MELD score	10.76 ± 4.36	9.33 ± 3.28	12.19 ± 4.89	0.126
Histopathologic findings				
NIS	3 (1-9)	3 (1-7)	3(1-9)	0.928
Fibrosis stage	0 (0-2)	0 (0-1)	0 (0-2)	1.000
Presence of fatty change	31 (81.6%)	15 (78.9%)	16 (84.2%)	1.000
Presence of Hemosiderin	20 (52.6%)	11 (57.9%)	9 (47.4%)	0.745
Prednisolone dosage (mg)	50 (20-120)	50 (20-60)	50 (20-120)	1.000

AST, aspartate transaminase; ALT, alanine transaminase; ALP, alkaline phosphatase; GGT, gamma glutamyl peptidase; INR, international normalized ratio; CTP, Child-Turcotte-Pugh; MELD, model for end-stage liver disease; NIS, necro-inflammatory score

Supplementary Table S2. Baseline characteristics and Laboratory, histopathologic findings and treatments between autoimmune hepatitis and primary biliary cholangitis

Variables	Total (N=40)	AIH (n=29)	PBC (n=11)	*P*-value
Age, years	62.5 (36-79)	65.0 (40-79)	53 (36-69)	0.013
Female sex (n,%)	35 (87.5%)	27 (93.1%)	8 (72.7%)	0.117
Laboratory findings				
Total bilirubin (mg/dL)	3.04 ± 4.17	3.7 ± 4.6	1.4 ± 2.3	0.004
Albumin (g/dL)	3.8 ± 0.6	3.6 ± 0.6	4.2 ± 0.4	0.006
AST (U/L)	383.8 ± 479.8	478.6 ± 507.8	133.9 ± 285.1	0.002
ALT (U/L)	279.4 ± 351.2	327.2 ± 330.3	153.4 ± 389.1	0.004
ALP (U/L)	135.4 ± 72.1	120.3 ± 65.1	175.0 ± 77.4	0.039
GGT (U/L)	265.9 ± 293.6	240.5 ± 312.7	332.9 ± 235.6	0.024
Creatinine (mg/dL)	0.8 ± 0.3	0.8 ± 0.4	0.7 ± 0.2	0.880
INR	1.1 ± 0.2	1.2 ± 0.2	1.1 ± 0.2	0.049
MELD score	10.4 ± 4.2	11.1 ± 4.3	8.6 ± 3.2	0.053
Histopathologic findings				
NIS score	8.5 ± 3.8	9.3 ± 3.7	6.3 ± 3.2	0.024
Fibrosis	1.5 (0-4)	2.0 (0-4)	1.0 (0-4)	0.166

AIH, autoimmune hepatitis; PBC, primary biliary cholangitis; AST, aspartate transaminase; ALT, alanine transaminase; ALP, alkaline phosphatase; GGT, gamma glutamyl peptidase; INR, international normalized ratio; CTP, Child-Turcotte-Pugh; MELD, model for end-stage liver disease; NIS, necro-inflammatory score

Supplementary Figure S1. Comparison of (A, B) treatment responses to steroid therapy and (C) relapse rates in patients with late responses, between the high portal T-cell infiltration group (the high group) and the low portal T-cell infiltration group (the low group). (D) Liver-related event-free survival (EFS) curves according to the high and low groups. **P*<0.05, ***P*<0.01.

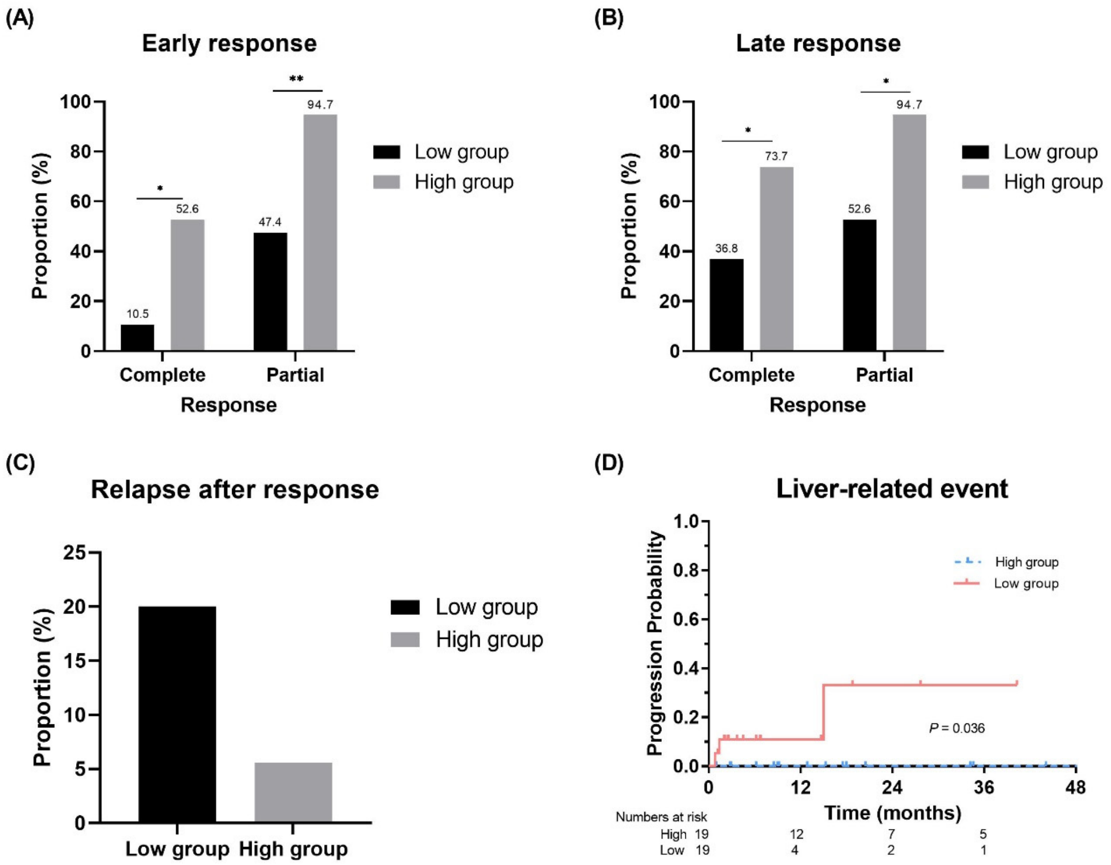

Supplementary Figure S2. Comparison of immunohistochemistry, including (A) CD20, (B) CD38, and (C) CD68, between AIH, PBC, and GVHD. AIH, autoimmune hepatitis; PBC, primary biliary cholangitis; GVHD, graft-versus-host disease. **P*<0.05, ***P*<0.01, ***P<0.001.

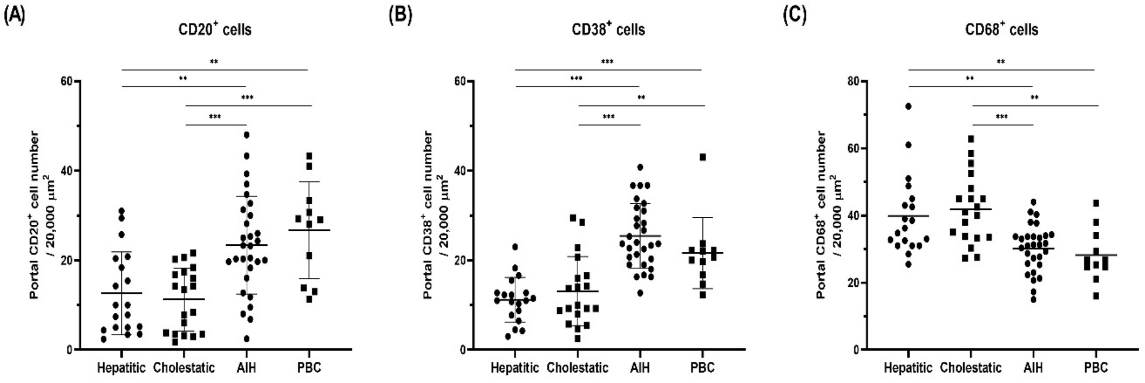

Supplement: Supplementary file 1 [file diagnostics-14-01745-s001.zip › diagnostics-3101259-supplementary.pdf]
